# Entrepreneurs’ Felt Responsibility for Constructive Change and Entrepreneurial Performance: A Moderated Mediation Model of Technology Action and Market Orientation

**DOI:** 10.3389/fpsyg.2021.751821

**Published:** 2021-12-22

**Authors:** Yuechao Du, Honghao Hu, Zhongming Wang

**Affiliations:** ^1^School of Management, Zhejiang University, Hangzhou, China; ^2^Global Entrepreneurship Research Center, Zhejiang University, Hangzhou, China

**Keywords:** felt responsibility for constructive change, entrepreneurial performance, technology action, market orientation, high-tech entrepreneurship

## Abstract

Drawing on self-determination theory, we examine the mechanism through which entrepreneurs’ felt responsibility for constructive change affects entrepreneurial performance and how market orientation affects the influencing mechanism. A questionnaire survey was conducted with 424 entrepreneurs in China. The results show that entrepreneurs’ felt responsibility for constructive change is positively related to technology action and entrepreneurial performance, and technology action mediates the relationship between entrepreneurs’ felt responsibility for constructive change and entrepreneurial performance. In addition, market orientation moderates the relationship between technology action and entrepreneurial performance such that the relationship is stronger when the market orientation is higher. Our findings suggest that when entrepreneur feel responsible for constructive change, they tend to take technology actions to achieve their goals and improve the long-term development of ventures. It is also important for entrepreneurs to hold a market orientation, which helps them be aware of changes in customer needs rather than blindly focusing on the use of the latest technologies. Our study is pioneering in exploring entrepreneurs’ felt responsibility for constructive change in the entrepreneurial context, advancing the research on entrepreneurship psychology.

## Introduction

In today’s dynamic and competitive environments, particularly since the coronavirus pandemic, entrepreneurs must take proactive actions to identify new opportunities and reallocate resources to reshape their business competitiveness through change to improve performance. There is growing recognition that entrepreneurs’ responsibility is critical in driving proactive action to adapt to the dynamic environment (Rooks et al., 2016) and dealing with potential crises in the organizations (Vallaster et al., 2019). Responsible entrepreneurs are those who “do what is right” and those who lead constructive change (Fuller et al., 2006). Previous research has identified entrepreneurs’ responsibility as an important psychological trait that could impact entrepreneurial success (Zhao and Seibert, 2006), and much attention has been dedicated to the external aspects of entrepreneurs’ responsibility, such as social corporate responsibility (Amaeshi et al., 2013; Tiba et al., 2019), but little research has addressed entrepreneurs’ internal responsibility, especially regarding its impact on the success of ventures (Fay and Frese, 2001; Wang and Zhao, 2017). Drawing on this line of research, we seek to explore how entrepreneurs’ internal responsibility (i.e., felt responsibility for constructive change) influences the success of entrepreneurship.

Our study focuses on entrepreneurial performance under the influence of entrepreneurs’ felt responsibility for constructive change, which implies that entrepreneurs’ proactive adoption of change and innovation behaviors, adherence to a long-term orientation, and responsibility for the outcomes of their actions could improve the performance (Saqib et al., 2018). Crant (2000) defines proactive action as the collection of a series of forward-looking, proactive, and change behaviors. This construct emphasizes that individuals who take action tend to influence organizational development in a self-determined manner by actively searching for opportunities and actively allocating resources (Dai et al., 2014). Due to the dynamics of the external environment, entrepreneurs must take proactive actions, including by paying attention to new trends in problem-solving processes and actively gathering the information and resources needed to implement innovation (Jensen et al., 2017). These processes are undoubtedly challenging and require entrepreneurs to actively overcome difficulties. Although some scholars believe that felt responsibility for constructive change is related to organizational performance, the behavioral mechanisms through which felt responsibility for constructive change affects entrepreneurial performance are unclear (Wallace et al., 2011).

According to self-determination theory, an individual’s intrinsic motivation can affect his or her behavior, thus influencing outcomes (Deci and Ryan, 2008; Uhl-Bien et al., 2014). Felt responsibility for constructive change can be viewed as a kind of intrinsic motivation that focuses on active participation in future achievement. Bakker et al. (2012) argue that taking proactive action derived from felt responsibility for constructive change is particularly important for entrepreneurs to access and identify external resources, thereby promoting entrepreneurial success (Hoang and Antoncic, 2003). One mechanism linking felt responsibility for constructive change to entrepreneurial performance is the transformation of responsibility for constructive change into a certain proactive action, such as technology action, which is related to performance in high-tech ventures.

**Figure 1:**
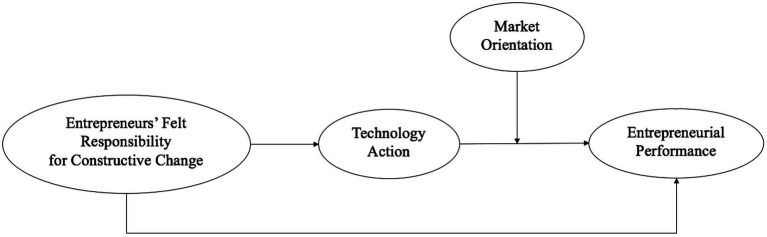
The theoretical model.

This study aims to elucidate the relationship between felt responsibility for constructive change and entrepreneurial performance which based on the mechanical framework of “motivation-behavior-performance” from self-determination theory. We argue that entrepreneurs’ felt responsibility for constructive change increases entrepreneurial performance through proactive action, especially in terms of the technology action of high-tech ventures. Moreover, we try to reveal the conditional effect of market orientation as extrinsic positive feedback, which could enhance the “motivation-behaviors-performance” path. The model discussed in this paper is shown in Figure 1. First, we review the relevant concepts, including felt responsibility for constructive change, technological action, market orientation, and entrepreneurial performance. Second, we include felt responsibility for constructive change as an independent variable and discuss its relationship to technology action and entrepreneurial performance. Finally, we focus on the moderating role played by market orientation.

## Literature Review

### The Perspective of Self-Determination Theory

Self-determination theory is a macro-level theory that is inspired by the early work of developmental, clinical, and humanistic psychologists. The theory employs a broad humanistic approach to motivation and personality development and offers a broad framework for understanding the factors that promote human motivation and behaviors, particularly the intrinsic motivation (Deci and Ryan, 2008). Prior studies have used self-determination theory in entrepreneurship research to discuss the relationship between the satisfaction of entrepreneurs’ three basic psychological needs – autonomy, competence, and relatedness – and their well-being (Al-Jubari et al., 2019; Shir et al., 2019; Lanivich et al., 2021). Although research on entrepreneurs’ felt responsibility research is growing (Baron, 2007; Elena et al., 2020), studies on explaining how and why entrepreneurs’ responsibility impacts entrepreneurial performance are still in infancy.

Self-determination theory proposes that individuals are all born with three innate psychological needs for autonomy, relatedness, and competence. When psychological needs are satisfied, an individual’s autonomous motivation will be promoted. Moreover, self-determination theory also emphasizes the importance of the intrinsic motivation to influence individual’s behavior to achieve high performance. The mechanism can be defined as the “motivation-behavior-performance” pathway (Deci and Ryan, 2008). Moreover, self-determination theory reveals the effect of external rewards on intrinsic motivation, especially in terms of the effect of positive feedback on intrinsic motivation (Deci et al., 2017). Although excessive external rewards have a negative effect on intrinsic motivation, appropriate external rewards and positive feedback can strengthen intrinsic motivation. Our theoretical model, which is based on self-determination theory, presents a possible mechanical framework of “motivation-behavior-performance” by highlighting the importance of felt responsibility for constructive change, which is intrinsically motivated, in benefiting entrepreneurial performance through the moderated mediation model of technology action and market orientation.

### Felt Responsibility for Constructive Change and Entrepreneurial Performance

Felt responsibility for constructive change is considered a psychological state in which an individual feels responsible for the work he or she is doing (Rachman, 1993; Morrison and Phelps, 1999). Prior research identifies both retrospective (reflecting on past behavior) and prospective (foreseeing future behavior) forms of felt responsibility (Cummings and Anton, 1990). Felt responsibility for constructive change is a kind of “assumed responsibility” rather than “assigned responsibility” (Fuller et al., 2006). Compared with “assigned responsibility,” felt responsibility for constructive change focuses on the future development of an organization. Relevant research mainly focuses on how felt responsibility for constructive change influences proactive employee behavior, such as voice behavior (Fuller et al., 2006; Tucker et al., 2008; Liang et al., 2012), extra-role behavior (Pearce and Gregersen, 1991), and organizational citizenship behavior (Choi, 2007). As felt responsibility for change is a proactive psychological state that relates to one’s initiative, individuals who feel personal responsibility for change tend to take ownership of the task process and feel accountable for the resulting outcome; thus, such individuals are more willing to assume risk in pursuing the goals and are more motivated to correct counterproductive procedures (Frese et al., 1997). Felt responsibility for constructive change also reflects the extent to which an individual believes that he or she can do a better job for a business, rather than simply performing the current task according to existing standards (Arain et al., 2019). Therefore, felt responsibility for constructive change is intentional and involves active participation in future achievement that extends beyond collaboration in existing work.

Much research has focused on the social corporate responsibility of entrepreneurs, which is the external aspect of entrepreneurs’ responsibility (Amaeshi et al., 2013; Tiba et al., 2019), but it has rarely focused on entrepreneurs’ internal responsibility, such as felt responsibility for constructive change. From the perspective of self-determination theory, felt responsibility for constructive change reflects the extent to which individuals internalize the value of constructive change (Bindl and Parker, 2011). Felt responsibility for constructive change is consistent with the concept of personal initiative. Accordingly, this type of responsibility involves considering the extent to which proactive action is useful in achieving a long-term orientation for entrepreneurs (Bateman and Crant, 1993; Rooks et al., 2016). Based on self-determination theory, an individual’s intrinsic motivation can affect his or her behavior, thus influencing outcomes (Deci and Ryan, 2008; Uhl-Bien et al., 2014). For an entrepreneur, felt responsibility for constructive change reflects an individual’s voluntary commitment to a sense of responsibility and forward-looking motivation that are not imposed by the organization. Entrepreneurs who feel more responsible for constructive change tend to take proactive actions and believe that the overall interests of their organizations are closely related to their own interests, allowing them to pursue long-term goals for firm development (López-Domínguez et al., 2013). Hence, compared to entrepreneurs with low responsibility for constructive change, those with high responsibility for constructive change will tend to act in ways that are more consistent with organizational goals, and they strongly internalize organizations’ long-term development as their assumed obligation, rather than assigned, obligation. In short, entrepreneurs’ felt responsibility for constructive change has an important impact on a venture’s performance.

H1: Entrepreneurs’ felt responsibility for constructive change has a positive impact on the performance of a venture.

### The Mediating Role of Technology Action

Technology action can be viewed as a certain kind of proactive action taken by entrepreneurs in high-tech ventures (McMullen and Shepherd, 2006), which involves specific behaviors associated with the usage of art of the technology, attentive search for breakthroughs in technology, and tracking the cutting-edge technology for long-term development (Choi and Williams, 2016). Technology action represents an entrepreneurs’ application of complex technologies in the development of new products (Filatotchev et al., 2009), which can be seen as an entrepreneurs’ initiative to adopt new ideas and propensity to utilize new technologies (Chen et al., 2014). Prior research indicates that technology action allows a firm to control its resources in an innovative and proactive manner and shows its willingness to take risks with such resources (Drummond et al., 2008; Williams and Lee, 2009; Kreiser and Davis, 2010). By adopting new technology, ventures can develop technical capabilities superior to those of their competitors to achieve higher customer value and customer satisfaction, long-term competitive advantage, and enterprise success. As a kind of proactive action, technology action reflects an entrepreneurs’ values and beliefs regarding the alertness to latest technology (Choi and Williams, 2016). Entrepreneurs’ technology action increases entrepreneurial performance by allocating resources needed to develop new capabilities and reinvigorate existing capabilities, thereby fostering an innovative mindset within the ventures. Technology action helps a venture perform better by guiding its utilization of resources in response to environmental signals before competitors (Campbell and Park, 2017; Covin et al., 2020). Therefore, technology action, which integrates sophisticated technology into a venture’s decision-making and organizing process, can significantly improve performance (Al-Henzab et al., 2018).

H2: Entrepreneurs’ technology action is positively related to entrepreneurial performance.

According to self-determination theory, entrepreneurs need to feel in control of their behaviors and goals (Cnossen et al., 2019). This sense of being able to take proactive action that will result in real change plays a major part in their perception of being self-determined (Wilson et al., 2003). Driven by felt responsibility for constructive change, entrepreneurs in high-tech industries tend to take technology actions to achieve their goals. Such a behavioral pattern affects their understanding and allocation of the internal and external resources or information of their enterprises, thus affecting firm performance. Therefore, entrepreneurs who feel a strong sense of responsibility for constructive change tend to emphasize changing the status quo and taking technology actions. These entrepreneurs are alert to the latest technologies and are willing to reallocate internal and external resources to integrate complex technologies as tools of the product development process (Adams et al., 2019).

H3: Entrepreneurs’ technology action mediates the positive relationship between felt responsibility for constructive change and entrepreneurial performance.

### The Moderating Role of Market Orientation

Entrepreneurship is a highly contextualized task, and entrepreneurs constantly adjust their intrinsic motivation and entrepreneurial actions based on the achievement of entrepreneurial goals and progress feedback (Frese et al., 1997; Omorede et al., 2015; Gielnik et al., 2020). Entrepreneurs with a market orientation believe that the competitive advantage of a venture mainly comes from the ability to create value for customers (Frank et al., 2016; Laukkanen et al., 2016). According to self-determination theory, extrinsic feedback will affect intrinsic motivation, which in turn influences behavior (Silva et al., 2014; Deci et al., 2017). Market orientation can be seen as a positively extrinsic feedback that can help entrepreneurs to obtain customer and market information to enhance their intrinsic motivation for entrepreneurship. These entrepreneurs pay close attention to changes in the market and meet the growing needs of customers to realize the value of a venture throughout the process. The key point of market orientation is to collect and use such information to create and deliver customer value (Martin et al., 2009; De Luca et al., 2010; Sommer, 2018). As they strengthen their market orientation, entrepreneurs gradually have increasing contact with customers, suppliers, and other stakeholders in the external environment and pay increasing attention to changes in competitors’ behavior (Boso et al., 2013). At this point, entrepreneurs monitor the market and technology dynamics in the external environment and search for possible knowledge sources (Morgan and Berthon, 2008). To improve the ability to meet customer needs, entrepreneurs require all staff to listen to the opinions of various groups by communicating with each other across departments and understanding the deficiencies of existing knowledge to meet customer needs and then increase input into external knowledge acquisition (Nouri and Ahmady, 2018). To develop new capabilities to meet future needs and create potential value, market-oriented entrepreneurs learn constantly to acquire knowledge and abilities related to the market, which will improve the performance of their ventures (Ho et al., 2018). When entrepreneurs take technology actions, having a stronger market orientation will strengthen the relationship between technology action and entrepreneurial performance.

H4: The relationship between technology action and entrepreneurial performance is moderated by entrepreneurs’ market orientation such that the relationship is stronger when market orientation is stronger.

The foregoing arguments explain the mediating role of technology action in the effect of felt responsibility for constructive change on entrepreneurial performance. In addition, it has been illustrated that market orientation acts as a moderator of the relationship between technology action and entrepreneurial performance. In combining these two effects, we propose that market orientation intensifies the mediating effect of technology action on the relationship between felt responsibility for constructive change and entrepreneurial performance, which can be represented by a moderated mediation model (Edwards and Lambert, 2007). Entrepreneurs with high market orientation in high-tech ventures are more likely to obtain customer and market information earlier, therefore, promoting entrepreneurial performance through technology action. Accordingly, the indirect effect of felt responsibility for constructive change on entrepreneurial performance could be stronger. We propose the following hypothesis. Figure 1 shows the theoretical model.

H5: Market orientation moderates the mediating effect of technology action on the relationship between felt responsibility for constructive change and entrepreneurial performance such that the mediating effect is stronger when the level of market orientation is high rather than low.

## Materials and Methods

Data for this study were collected from a survey of innovation behavior and entrepreneurial performance of high-tech ventures in China. First, 21 ventures in Zhejiang Province were selected to participate in a prequestionnaire survey. After revising and improving the questionnaire based on the questions and suggestions raised by the interviewees during the pre-survey, we conducted a formal questionnaire survey with 448 entrepreneurs in high-tech industries, such as the electronics, biopharmaceutical, and new material industries from Zhejiang, Shanghai, and Jiangsu Provinces. The participants were entrepreneurs with a comprehensive grasp of organizational characteristics, such as the strategy and performance of ventures, and their answers can reflect the real state of their ventures. The survey was conducted in two rounds. The first survey measured demographic variables and items on felt responsibility for constructive change, technology action, and control variables. The second survey, conducted 6 months later, measured market orientation and entrepreneurial performance. Complete and valid questionnaires from 424 entrepreneurs were obtained, and the effective questionnaire rate was 94.6%.

### Measures

The original survey scales used in this paper were all obtained from previous empirical studies. First, we used the translation-back-translation procedure to translate the questionnaire (Reynolds et al., 1993). To tailor the questionnaire to the Chinese cultural context, we appropriately modified the questions *via* discussions with experts. However, only a few changes to the original survey scales were needed, and some items were removed to make the questionnaire more concise.

#### Felt Responsibility for Constructive Change

We used Morrison and Phelps’ (1999) five-item measure to evaluate one’s feelings of responsibility for constructive change. Entrepreneurs responded using a 5-point response scale ranging from 1 = “strongly disagree” to 5 = “strongly agree.” Example items include “I feel a personal sense of responsibility to bring about change at work” and “I feel obligated to try to introduce new procedures where appropriate.” Cronbach’s alpha for this measure was 0.81.

#### Technology Action

We used a four-item scale developed by Gatignon and Xuereb (1997). Entrepreneurs responded using a 5-point response scale ranging from 1 = “strongly disagree” to 5 = “strongly agree.” A sample item is “We use sophisticated technologies in our new product development.” Cronbach’s alpha for this measure was 0.88.

#### Market Orientation

Market orientation was measured with the scales originally developed by Narver and Slater (1990) and adapted for our research. All items used to measure these constructs are based on 5-point Likert scales, and the scale consists of six items measuring an entrepreneur’s market orientation. A sample item is “We constantly monitor our level of commitment and orientation in serving customers’ needs.” Cronbach’s alpha for this measure was 0.84.

#### Entrepreneurial Performance

We used the scale developed by Li and Atuahene-Gima (2001) measuring profit growth, overall efficiency of operations, sales growth, market share growth, and firm’s overall reputation. A sample item is “Relative to your principal competitors, rate sales growth of your firm over the last three years”. The Cronbach’s alpha for this measure was 0.87.

#### Control Variables

Following previous research (Fuller et al., 2006; Covin et al., 2020), we controlled for individual-level variables, including gender, age, education, and entrepreneurial experience, and firm-level variables, including firm size, and firm age. For entrepreneurial experience, entrepreneurs with entrepreneurial experience were coded as 1, while those without entrepreneurial experience were coded as 0. Education was decomposed into two dummy variables. For edu_1, those with a master’s degrees were coded as 1, others are coded as 0. For edu_2, those with a doctoral degrees were coded as 1, others are coded as 0. For firm age, the number of years for which the firm had existed was used. Firm size was calculated from the natural logarithm of the number of employees.

### Common Method Bias

Data were collected by a self-report questionnaire, and one questionnaire was completed by the same respondent, raising issues regarding common method variance (CMV). Harman’s one-factor test was thus applied to examine CMV. Four factors were extracted, and the first factor could explain only 28.46% of the variance, indicating that CMV was not a serious problem in our survey.

## Results

### Descriptive Statistics

The descriptive statistics and correlations of the variables examined in our study are presented in Table 1. We also calculated variance inflation factors (VIFs) to examine the multicollinearity of the variables. The VIFs are all below 10 (1.39 for felt responsibility for constructive change, 1.46 for technology action, and 1.23 for market orientation), denoting that no serious multicollinearity existed in the regression model (Hair et al., 2010).

**Table 1:** Descriptive statistics and intercorrelations of variables.

S. No	Variables	*M*	*SD*	1	2	3	4	5	6	7	8	9	10
1.	Gender	0.12	0.33										
2.	Age	36.66	3.85	0.26^*^									
3.	Edu_1	0.32	0.47	0.12	−0.14^**^								
4.	Edu_2	0.60	0.49	0.04	0.19^**^	−0.73^**^							
5.	EE	0.85	0.36	−0.07	0.31^*^	0.02	0.03						
6.	FS	3.96	0.60	0.24	0.09	0.03	−0.13	−0.18^**^					
7.	FA	4.53	1.33	−0.04	0.06	−0.02	0.04	0.01	0.05				
8.	FRCC	4.61	0.47	0.03	−0.01	−0.05	−0.01	0.01	−0.01	0.09			
9.	TA	4.37	0.46	0.04	0.06	0.03	0.06	0.01	−0.02	0.06	0.51^**^		
10.	MO	3.66	0.73	0.12^**^	0.04	−0.03	−0.02	0.04	−0.02	−0.06	0.34^**^	0.40^**^	
11.	EP	4.11	0.57	0.14^*^	0.01	−0.01	−0.05	−0.02	0.03	0.01	0.42^**^	0.55^**^	0.53^**^

* *p < 0.05*;

** *p < 0.01*.

*EE = Entrepreneurial experience; FS = Firm size; FA = Firm age; FRCC = Felt responsibility for constructive change; TA = Technology action; MO = Market orientation; EP = entrepreneurial performance. n = 424. Internal consistency coefficients are reported in bold on the diagonal*.

### Confirmatory Factor Analysis

We conducted a confirmatory factor analysis to validate the measures (see Table 2). The fit indexes suggest a good fit for our hypothesized four-factor model with *χ*2/*df* = 2.568, CFI = 0.979, TLI = 0.918, RMSEA = 0.080, and SRMR = 0.025. The observed items significantly load on the expected latent factors. To further test our measures, we compared the hypothesized four-factor model to three alternative models: (1) a three-factor model with felt responsibility for constructive change and market orientation loading on one latent factor and with the other constructs loading on their own respective factors, with *χ*2/*df* = 6.648, *p* < 0.01, indicating a worse fit than the hypothesized model; (2) a three-factor model with felt responsibility for constructive change and technology action loading on one latent factor and other variables loading on their own factors, with *χ*2/*df* = 4.530, *p* < 0.01, indicating a worse fit than the hypothesized model; (3) a three-factor model with market orientation and technology action loading on one latent factor and other variables loading on their own factors, with *χ*2/*df* = 4.986, *p* < 0.01, indicating a worse fit than the hypothesized model; and (4) a two-factor model with felt responsibility for constructive change, technology action and market orientation together loading on one factor, with *χ*2/*df* = 7.289, *p* < 0.01, also indicating a worse fit than the hypothesized two-factor model. The results therefore provide support for the distinctiveness of the four constructs as hypothesized.

**Table 2:** Confirmatory factor analysis.

Factor structure	*χ*^2^/df	RMSEA	CFI	TLI	SRMR
Four-factor model (felt responsibility for constructive change; technology action; market orientation; entrepreneurial performance)	2.568	0.080	0.979	0.918	0.025
Three-factor model (combining felt responsibility for constructive change and market orientation together)	6.648	0.111	0.728	0.688	0.105
Three-factor model (combining felt responsibility for constructive change and technology action together)	4.530	0.094	0.830	0.805	0.095
Three-factor model (combining market orientation and technology action together)	4.986	0.100	0.808	0.779	0.082
Two-factor model (combining felt responsibility for constructive change, technology action together)	7.289	0.124	0.693	0.652	0.106
One-factor model (combining all items into one factor)	8.592	0.137	0.627	0.58	0.101

### The Main Effects of Felt Responsibility for Constructive Change and the Mediating Effects of Technology Action and Market Orientation

Multiple regression analyses were used to test the hypotheses. As shown in Table 3, we use SPSS PROCESS macro (Hayes, 2017) to test the mediating mechanism. In Model 1, all of the control variables are regressed on technology action, while in Model 3, control variables are regressed on entrepreneurial performance. In Model 4, the control variables and felt responsibility for constructive change are regressed on entrepreneurial performance. The result reveals a significant relationship between entrepreneurs’ felt responsibility for constructive change and entrepreneurial performance (*b* = 0.51, *p* < 0.01). Hypothesis 1 is therefore supported. In Model 2, the control variables and independent variable (felt responsibility for constructive change) are regressed on technology action. The result shows that entrepreneurs’ felt responsibility for constructive change is significantly related to technology action (*b* = 0.49, *p* < 0.01). In Model 5, felt responsibility for constructive change and technology action are simultaneously regressed on entrepreneurial performance. The result indicates that technology action is significantly related to entrepreneurial performance (*b* = 0.54, *p* < 0.01). Hypothesis 2 is thus supported. In Model 5, entrepreneurs’ felt responsibility for constructive change is also significantly related to entrepreneurial performance (*b* = 0.22, *p* < 0.01), which indicates that technology action partly mediates the relationship between entrepreneurs’ felt responsibility for constructive change and entrepreneurial performance. Hypothesis 3 is thus supported.

**Table 3:** Regression results of mixed model.

	Technology action		Entrepreneurial performance			
	M1	M2	M3	M4	M5	M6
**CV**						
Gender	0.06^*^	0.02	0.29^**^	0.25^**^	0.23^*^	0.18^*^
Age	0.01	0.01	−0.01^*^	−0.01	−0.01	−0.01
Edu_1	−0.09	0.02	−0.21	−0.11	−0.12	−0.08
Edu_2	−0.14	−0.06	−0.23	−0.15	−0.12	−0.08
EE	−0.01	−0.02	0.01	0.01	0.01	−0.01
FS	−0.03	−0.02	0.01	0.01	0.02	0.02
FA	0.02	0.01	0.01	−0.01	−0.01	0.01
**IV**						
FRCC		0.49^**^		0.51^**^	0.22^**^	0.15^**^
**Me**						
TA					0.54^**^	0.45^**^
**Mo**						
MO						0.24^*^
**Interaction**						
TA * MO						0.15^**^
*R* ^2^	0.02	0.27	0.04	0.20	0.36	0.46
*F* ^2^	1.03	18.99^**^	2.19^*^	13.34^**^	25.99^**^	31.82^**^

* *p < 0.05*;

** *p < 0.01*.

*EE = Entrepreneurial experience; FS = Firm size; FA = Firm age; FRCC = Felt responsibility for constructive change; TA = Technology action; MO = Market orientation; EP = entrepreneurial performance*.

Table 4 presents the result for the mediating effect. The total effect of felt responsibility for constructive change on entrepreneurial performance is significantly positive (*b* = 0.51, *p* < 0.01). Table 4 also shows the direct effect of felt responsibility for constructive change on entrepreneurial performance that excludes the indirect effect of the mediator. Furthermore, we adopted bootstrap methods to test the mediating role of technology action, which takes the indirect effect into consideration (Shrout and Bolger, 2002). The mediating effect was tested with the expectation that the indirect effect should not be zero (MacKinnon et al., 1995). The result shows an indirect effect of felt responsibility for constructive change on entrepreneurial performance *via* technology was of 0.29 (95% CI [0.21, 0.38]).

**Table 4:** The regression analysis of the mediating effect.

Effect	B	SE	LLCI	ULCI
Direct effect of X on Y	0.22^**^	0.06	0.11	0.33
Indirect effect of X on Y	0.29^**^	0.04	0.21	0.38
Total effect of X on Y	0.51^**^	0.05	0.40	0.61

** *p < 0.01*.

### The Moderating Role of Market Orientation

**Figure 2:**
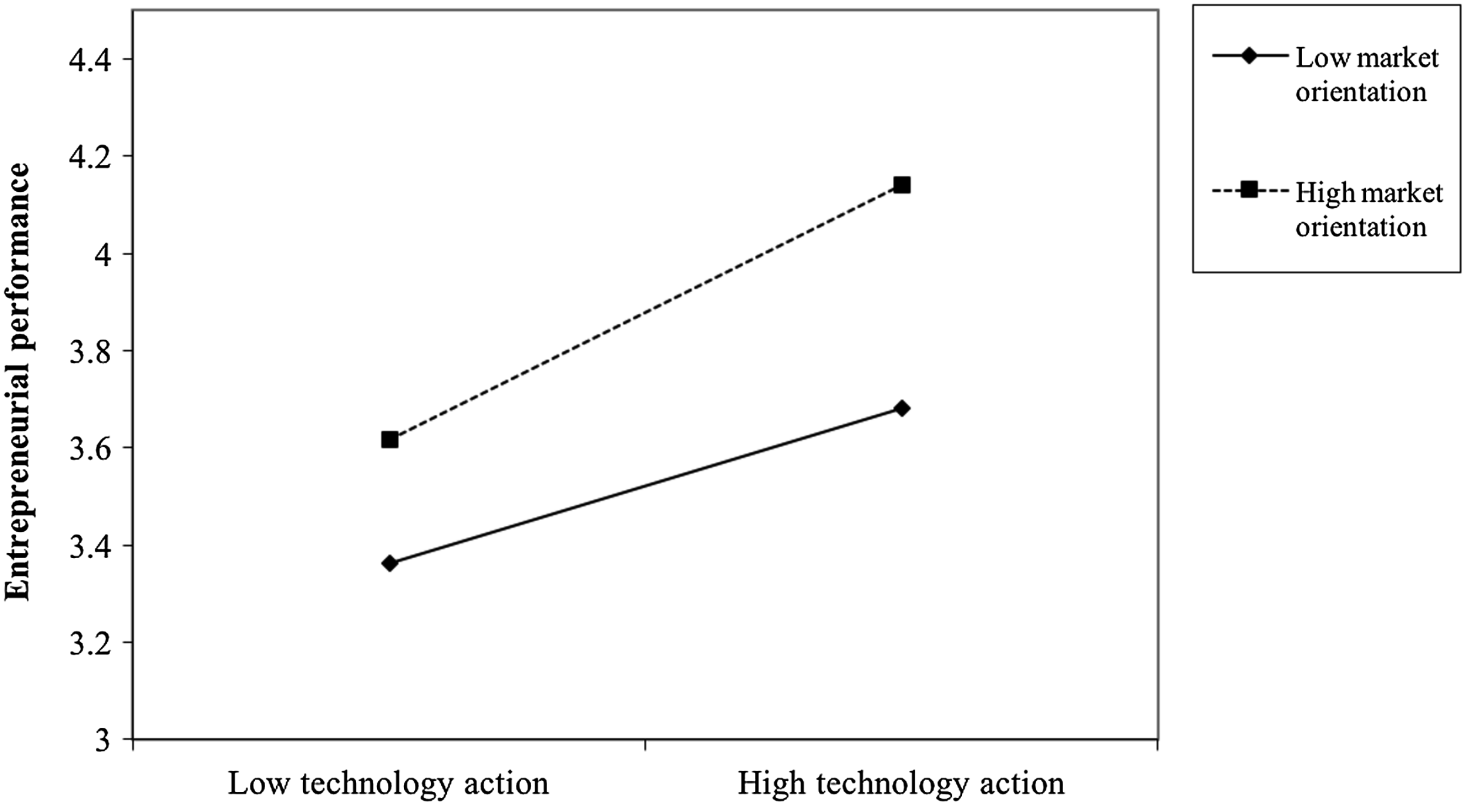
Interactive effects of market orientation and technology action on entrepreneurial performance.

The interaction between technology action and entrepreneurial performance was tested. The results of Model 6 in Table 3 show the interaction between technology action and market orientation significantly predict entrepreneurial performance (*b* = 0.15, *p* < 0.01). Thus, Hypothesis 4 is supported. We also tested the moderating effects of market orientation on the relationship between technology action and entrepreneurial performance. Furthermore, the conditional effect of technology action on entrepreneurial performance for different values of the moderator (−1 *SD* as Low; +1 *SD* as High) is shown in Table 5. The indexes of moderated mediation are presented in Table 6. The index for entrepreneurial performance is 0.08, with confidence intervals excluding zero. Thus, Hypothesis 5 is supported. To further show the moderating effect of market orientation, we plotted the effect of technology action on entrepreneurial performance on the basis of a high versus low level of market orientation. The plot (see Figure 2) indicates that technology action has a stronger effect on entrepreneurial performance when the market orientation is strong rather than weak.

**Table 5:** The conditional effect of technology action on entrepreneurial performance.

Moderator	Effect	SE	LLCI	ULCI
Low	0.17	0.05	0.09	0.28
Mean	0.23	0.04	0.16	0.32
High	0.29	0.04	0.21	0.38

**Table 6:** Index of moderated mediation.

Outcome	Index	SE	LLCI	ULCI
Entrepreneurial performance	0.08	0.03	0.03	0.14

## Discussion

Our study shows that entrepreneurs’ felt responsibility for constructive change has a positive effect on entrepreneurial performance through technology action. Furthermore, entrepreneurs’ market orientation moderates the mediating effect of technology action on the relationships between felt responsibility for constructive change and entrepreneurial performance, such that the mediating effect is stronger when the level of market orientation is high rather than low.

### Theoretical Implications

The study extends our knowledge of the relationship between entrepreneurs’ felt responsibility for constructive change and entrepreneurial performance, its underlying mechanism, and conditional limits, thereby contributing to entrepreneurship psychology research in notable ways. Previous studies have mainly focused on the employee level, emphasizing the impact of employees’ felt responsibility for constructive change on voice behavior, innovation, and organizational performance (Fuller et al., 2006; Liang et al., 2012). Based on former research, this study extends the research scope of felt responsibility for constructive change to entrepreneurs and explores the influence of this sense of responsibility on entrepreneurial performance by using self-determination theory to build a linkage. For entrepreneurs, the responsibility they feel for constructive change involves a voluntary personal commitment to responsibility and proactive motivation that is not imposed by an organization. Entrepreneurs who feel strong responsibility for constructive change tend to adjust their action mode according to the future development of their organizations.

This study also increases confidence in the explanatory power of self-determination theory in contexts of entrepreneurship, which may expand the scope of the theory, by empirically showing how entrepreneurs’ felt responsibility for constructive change promotes entrepreneurial performance through technology action. This study relies on self-determination theory and proposes that entrepreneurs need to have a sense of control over their behaviors and goals, which plays an important role in choosing behavioral patterns. Entrepreneurs in high-tech industries driven by a sense of responsibility for constructive change are more likely to take technology actions to help achieve their goals, and they emphasize changing the status quo. Since technical competence and knowledge can improve product design and quality, entrepreneurs show greater enthusiasm for and confidence in applying cutting-edge technologies as tools in the product development process, participating in the innovation process, and improving product functions (Autio et al., 2014; Van Minh et al., 2017). Moreover, this study reveals the conditional effect of market orientation as positive external feedback, which could enhance the “motivation-behaviors-performance” path.

### Practical Implications

In the post-epidemic era, to deal with various challenges, entrepreneurs should develop a sense of responsibility for constructive change to adapt to the dynamic environment. The entrepreneurial performance of high-tech ventures often must be based on the output of innovation. Innovation usually involves an act of positive change, which is defined as “self-initiated, long-term oriented proactive action and persistence in the face of barriers and obstacles that need to be overcome” (Hagedoorn, 1996; Bledow et al., 2009). As Ghitulescu (2013) states, people who actively take proactive actions are relatively unconstrained by environmental forces and instead influence the environment through change behavior as well as bearing the consequences of change. Entrepreneurs need to take proactive actions to actively explore opportunities and persevere in exploiting them to allocate resources while bearing the associated consequences. Entrepreneurial opportunities can be viewed as potentially profitable but hitherto unexplored projects (Casson and Wadeson, 2007). Opportunity identification and resource allocation are proactive processes whereby the entrepreneur abandons the original model and combines information in new ways. In addition to achieving proactive creation during the idea generation phase, entrepreneurs must also take responsibility for the outcome of constructive changes in the implementation of the innovation, especially for high-tech ventures in the post-crisis era.

Driven by felt responsibility for constructive change, entrepreneurs in high-tech industries tend to be alert to the cutting-edge technologies. Such a behavioral pattern affects an entrepreneur’s understanding and allocation of the internal and external resources or information of an enterprise, thus affecting entrepreneurial performance (Saqib et al., 2018). Meanwhile, for entrepreneurs of high-tech industries in China, a market orientation is important in helping them be aware of changes in customer needs rather than blindly focusing on the use of the latest technologies. Entrepreneurs with a market orientation pay close attention to changes in the market and to meeting the growing needs of customers to realize the value of the ventures in the process, thus earning competitive advantages in the market.

### Limitations and Future Research

This study has several limitations that future research could address. Our study sample includes only high-tech ventures based in the Yangtze River Delta. Although using such a sample can better control the influence of regional and industrial factors, which is conducive to improving the internal validity of the research, it also restricts the external validity of our results to some extent. Whether the relevant conclusions of this study can be extended to ventures in other regions and other industries may need further confirmation based on more studies. Therefore, subsequent studies on a wider range of geographical areas and industries can be conducted to test the findings of this study.

Our survey examined the impact of only individual-level variables on entrepreneurial performance. To enrich theories and knowledge of felt responsibility for constructive change and performance at the team level, future research should adopt a multilevel research design to explore the impact of environmental factors at the team level (such as team innovation climate) on the relationship between entrepreneurs’ felt responsibility for constructive change and the performance of their ventures.

## Conclusion

This study examined how entrepreneurs’ felt responsibility for constructive change affects entrepreneurial performance. Our results suggest that technology action mediates the positive relationship between felt responsibility for constructive change and entrepreneurial performance; that is, entrepreneurs’ felt responsibility for constructive change positively influences entrepreneurial performance through the technology actions they take. We also found that entrepreneurs’ market orientation moderates the relationship between technology action and entrepreneurial performance. In particular, a strong market orientation strengthens the positive relationship between technology action and entrepreneurial performance.

## Data Availability Statement

The original contributions presented in the study are included in the article/supplementary material, and further inquiries can be directed to the corresponding author.

## Author Contributions

All authors listed have made a substantial, direct and intellectual contribution to the work, and approved it for publication.

## Funding

This research was supported by Social Science Foundation of Fujian Province (FJ2021B157).

## Conflict of Interest

The authors declare that the research was conducted in the absence of any commercial or financial relationships that could be construed as a potential conflict of interest.

## Publisher’s Note

All claims expressed in this article are solely those of the authors and do not necessarily represent those of their affiliated organizations, or those of the publisher, the editors and the reviewers. Any product that may be evaluated in this article, or claim that may be made by its manufacturer, is not guaranteed or endorsed by the publisher.
